# Phase Equilibria of the Ag-Al-Au Ternary System and Interfacial Reactions in the Au-*x*Ag/Al Couples at 450 °C

**DOI:** 10.3390/ma16227196

**Published:** 2023-11-16

**Authors:** Mavindra Ramadhani, Andromeda Dwi Laksono, Chien-Lung Liang, Chiao-Yi Yang, Kuo-Jung Chen, Yee-Wen Yen, Hsien-Ming Hsiao

**Affiliations:** 1 Department of Materials Science and Engineering, National Taiwan University of Science and Technology, Taipei 10633, Taiwan; mavindra@its.ac.id (M.R.); andromeda@lecturer.itk.ac.id (A.D.L.); clliang@mail.ntust.edu.tw (C.-L.L.); sandy004100@gmail.com (C.-Y.Y.); m10604318@mail.ntust.edu.tw (K.-J.C.); 2 Sustainable Electrochemical Energy Development Center (SEED Center), National Taiwan University of Science and Technology, Taipei 10633, Taiwan; 3 National Atomic Research Institute, Taoyuan 32546, Taiwan; hmhsiaon@nari.org.tw

**Keywords:** Ag-Al-Au ternary system, solid-state reaction couple, Au-*x*Ag/Al system, isothermal section, intermetallic compound (IMC), reaction controlled

## Abstract

The phase equilibria of the Ag-Al-Au ternary system and the solid-state reaction couple for the Au-*x*Ag/Al system were investigated isothermally at 450 °C. By investigating the Ag-Al-Au ternary system and its isothermal section, this study aims to provide a clearer understanding of the phase stability and interfacial reactions between different phases. This information is crucial for designing materials and processes in electronic packaging, with the potential to reduce costs and improve reliability. There were seven single-phase regions, thirteen two-phase regions, and six three-phase regions, with no ternary intermetallic compound (IMC) formed in the isothermal section of the Ag-Al-Au ternary system. When the Au-25 wt.% Ag/Al couple was aged at 450 °C for 240–1500 h, the AuAl_2_, Au_2_Al, and Au_4_Al phases formed at the interface. When the Ag contents increased to 50 and 75 wt.%, the Ag_2_Al, AuAl_2_, and Au_4_Al phases formed at the interface. When the aging time increased from 240 h to 1500 h, the total IMC thickness in all Au-*x*Ag/Al couples became thicker, but the types of IMCs formed at the interface did not change. The total IMC thickness also increased with the increase in the Ag content. When the Ag content was greater than 25 wt.%, the Au_2_Al phase was converted into the Ag_2_Al phase. The IMC growth mechanism in all of the couples followed a reaction-controlled process.

## 1. Introduction

Electronic products, especially consumer electronics, play an essential part in humans’ life. Technological advancements have increased the demand for lighter, thinner, shorter, and smaller electronic products. The interconnection technology between integrated circuit (IC) chips and substrates in electronic packaging has become more important nowadays. The wire bonding technique is the most common type of interconnection between an IC chip and lead frame, using a metal wire with a 15–50 μm diameter to connect the chip and lead frame with thermocompression bonding, ultrasonic bonding, or thermosonic bonding methods. This technique is a very cost-effective and flexible interconnection technique for the first level of electronics packaging. This research contributes to the field of materials science and electronic packaging technology, potentially offering solutions to industry challenges related to material costs and interfacial reactions. Moreover, this research has several applications, such as electronic packaging, which is the primary application of this research, phase diagram understanding, which is essential for predicting the stability of different phases, and materials selection for Ag or Ag-Au alloys as replacements for Au wire bonding. In real electronic packaging, wire bonding is used for connecting ICs to substrates or lead frames. The primary goals are to ensure reliable electrical connections, good thermal performance, and mechanical strength.

Au is one of the primary materials used in wire bonding because of its high reliability, good corrosion resistance, and superior electrical conductivity characteristics. Al is generally used as the pad material deposited on an IC’s surface because of its low electrical resistivity, good thermal conductivity, and inexpensive characteristics. Based on the positive properties mentioned above, Au and Al are widely used in the electronic packaging industry. However, the ever-increasing price of Au is a big issue in conventional Au wire bonding technology at the first level of electronic packaging [1]. Additionally, when electronic devices are under operation, heat is generated, which further causes interdiffusion between the Au wire and Al pad, and an Au-Al intermetallic compound (IMC) with a brittle nature forms at the Au/Al interface. Moreover, Ag, with a high melting temperature (960 °C), is considered as one of the ideal packaging materials [2]. High electrical resistance was discovered in the IMCs, and the growth rate of Au-Al IMCs was ten times that of the Cu-Al IMCs [3]. When the Au-Al IMCs form at the interface, their thickness increases with the increase in the operational period. The thicker IMC would increase the electrical resistivity and decrease the mechanical performance at the Au/Al interface [4]. Thus, several materials have been investigated to replace the Au wire to reduce the cost and improve the reliability of the Au/Al joint. Solder joints are subjected to varied stress cycle circumstances in the electronic packaging service [5]. The physical properties of pure Ag, such as the melting point, thermal conductivity, electrical conductivity, hardness, and Young’s modulus, are similar to pure Au or even better [6]. The Ag-Au system is an isomorphous system. This means that Ag and Au can be completely mixed in the solid phase without any IMCs. This is involved to high latent heat phase change-based electronic packaging materials [7]. Meanwhile, the price of Ag is one hundredth that of Au [8]. Thus, Ag or Ag-Au alloys have high potential as candidates to replace Au wire.

Phase diagrams are powerful tools to understand and explain the phase stability and interfacial reactions among different phases [9,10]. From the perspective of the Ag-Au wire bonding onto the Al pad, a clearer understanding of the Ag-Al-Au ternary system is necessary. The constituent binary phase diagrams for the Ag-Al [11], Ag-Au [8], and Al-Au [12] systems have been reported. Prince et al. investigated the Ag-Al-Au ternary system and reported the isothermal section of the Ag-Al-Au ternary system at 500 °C [13]. They found considerable solubility of Ag in the AlAu_4_ phase, and no ternary IMC was found in this system. In other research conducted by Petzow [14], it was also revealed that there are Ag-rich phases in (Au, Ag)_4_Al, which are formed in the Ag-Al-Au phase diagram. In this work, the phase equilibria of the Ag-Al-Au ternary system and interfacial reaction in the Au-*x*Ag/Al reaction couples were investigated at 450 °C. An arc melting furnace was used to prepare different compositions of the Ag-Al-Au ternary and Au-Ag binary alloys. The solid/solid reaction couple of the Au-*x*Ag/Al system was aged at 450 °C for 240 to 1500 h. The type, morphology, thickness, and growth kinetics of the IMCs that formed between the Au-*x*Ag alloys and Al were investigated. The isothermal section of the Ag-Al-Au ternary system was used to explain the formation of IMCs and to describe the reaction path in the Au-*x*Ag/Al reaction couples.

## 2. Experimental Procedures

### 2.1. Phase Equilibria of the Au-Sn-Zn Ternary System

In order to investigate the phase equilibria of the Ag-Al-Au ternary system, seventeen Ag-Al-Au alloys with different compositions were designed and prepared from the pure Ag, Al, and Au shots of over 99.9 wt.% purity (supplied by Alfa Aesar, Haverhill, MA, USA). This alloy is used to establish the isothermal section of the Ag-Al-Au ternary at 450 °C, which requires systematically exploring different compositions to create a comprehensive phase diagram. The compositions of the alloys produced are listed in Table 1. Each alloy specimen had a total mass of 1.0 g. The constituent pure element shots were cleaned with acetone, hydrochloric acid, and alcohol sequentially to remove the oxide layer and impurities before the following alloy preparation. The arc melting furnace (Miller, Gold Star 602; Appleton, WI, USA) was used to prepare the Ag-Al-Au ternary ingots with designed compositions. In order to make sure all of the elements were well mixed during the melting process, each sample was melted at least three times by turning it over and then arc melting it again. After the arc melting, all of the alloys were encapsulated in a quartz tube by using argon as a protective gas in a vacuum of 0.1 Pa. The sample tube was thermally aged in a furnace at 450 °C for 1440 h to reach an equilibrium state. The alloy ingot was quenched in icy water and removed from the quartz tube. Each alloy was cut into halves.

One half of the alloy specimen was treated metallographically. An optical microscope (OM; Olympus, BX-51M; Tokyo, Japan) and scanning electron microscope (SEM; Hitachi, TM 3000; Tokyo, Japan) were used for the microstructural examination. An energy-dispersive spectrometer (EDS; Bruker, Quantax 70; Billerica, Berlin, Germany) equipped within an SEM and electron probe microanalyzer (EPMA; Jeol JSM-8200; Osaka, Japan) were used for the compositional analysis. To obtain a diffraction pattern, the second half of the alloy specimen in bulk form was analyzed using an X-ray diffractometer (XRD, Bruker, D2 Phaser; Germany). Using the International Centre for Diffraction Data (ICDD) database [15] to compare the diffraction angles, the phases of the alloys were identified. To identify the phases present in each equilibrated alloy, the microstructures, elemental composition, diffraction patterns, and constituent binary phase diagrams [8,11,12] were collectively examined.

### 2.2. Interfacial Reactions in the Au-xAg/Al Reaction Couple

The high-purity (99.95 wt.%) Au and Ag elements were used in the arc melting furnace (Miller, Gold Star 602; Appleton, WI, USA) to prepare the Au-*x*Ag (*x* = 25, 50, and 75 wt.%) alloy. Each alloy mixture was melted at least 5 times to prevent heterogenization and ensure that all elements mixed completely. Each alloy ingot was encapsulated in a quartz tube evacuated under 0.1 Pa and placed in a furnace at 900 °C for 72 h to let the alloy homogenize. Then, the alloy was taken out and quenched in ice water. The thickness of the Al foil used in this study was 2 mm. The homogenized Au-*x*Ag alloy and Al foil were cut into 5.0 × 5.0 × 2.0 mm^3^ sizes and pressed to give them flat shape. The Au-*x*Ag alloy and Al foil were given a metallographic treatment to obtain a clean surface. After being polished, the specimens were cleaned using an ultrasonic vibration cleaner for 15 min to remove the remaining Al_2_O_3_ particles and other impurities on the surface. A rosin mildly activated (RMA) flux (Magna 51 flux, Troy, MI, USA) was dipped between the Au-*x*Ag alloys and Al to increase the solderability and remove the oxide. The Au-*x*Ag alloy and Al were sandwiched together using two stainless steel screws to form the Au-*x*Ag/Al reaction couple. This process helps the bonding by applying a press to produce a reaction couple joint. The sandwich was sprayed with boron nitride to avoid a reaction between the reaction couple and stainless steel. The Au-*x*Ag/Al reaction couple fixed with screws was placed in a quartz tube and vacuum-treated under 0.1 Pa. Then, the Au-*x*Ag/Al reaction couple in the quartz tube was put in the oven and aged at 450 °C for 240, 480, 600, 1000, and 1500 h. After aging, the couple was taken out and cooled down at room temperature. The reaction couple was mounted using a mounting press machine (SimpliMent 1000, Buehler; Plymouth, MN, USA), and metallographic treatment, including grinding and polishing, was performed to obtain a smooth surface on the cross section.

An OM and field emission scanning electron microscope (FE-SEM; Jeol-7900F; Tokyo, Japan) were used to observe the microstructure of the interfacial reaction morphology. The quantitative compositional analysis of the IMC was performed using SEM-EDS and EPMA. The elemental compositions obtained from the EDS and EPMA were compared with the constituent phase diagrams (Au-Ag, Au-Al, and Ag-Al binary phase diagrams) to determine the phases. The image analysis software, ImageJ windows version with Java 8, was used to measure the average IMC thickness at the interface. The IMC thickness was determined by dividing the area of a selected region by the linear length of the measurement area. Each IMC thickness presented for the reaction couples was averaged over five different areas to obtain reliable data.

## 3. Results and Discussion

### 3.1. Phase Equilibria of the Ag-Al-Au Ternary System at 450 °C

**Figure 1:**
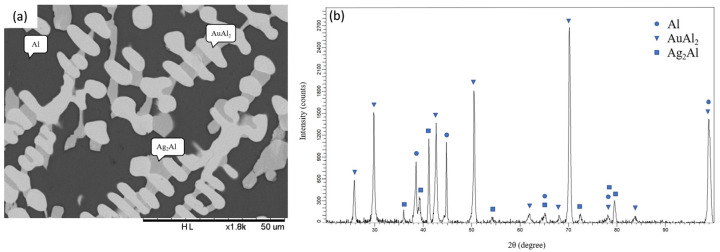
(**a**) BEI micrograph and (**b**) XRD pattern of the Ag-80 at.%Al-10 at.%Au alloy (Alloy 1) aged at 450 °C for 1440 h.

**Figure 2:**
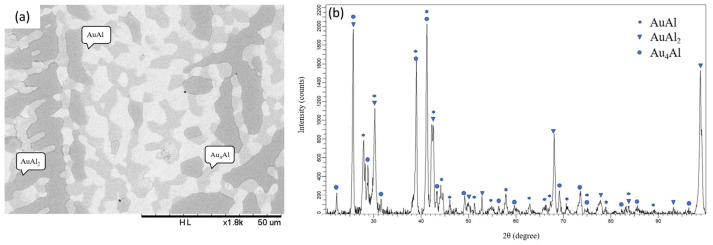
(**a**) BEI micrograph and (**b**) XRD pattern of the Ag-50 at.%Al-40 at.%Au alloy (Alloy 6) aged at 450 °C for 1440 h.

**Figure 3:**
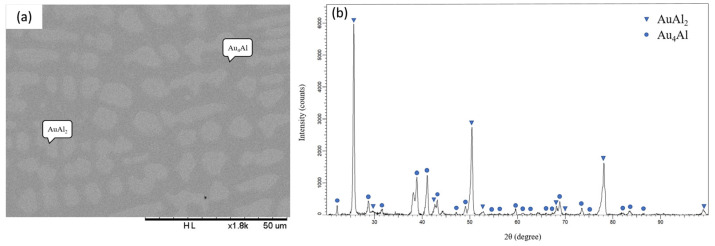
(**a**) BEI micrograph and (**b**) XRD pattern of the Ag-40 at.%Al-20 at.%Au alloy (Alloy 8) aged at 450 °C for 1440 h.

Figure 1a presents the backscattered electron image (BEI) micrograph of Alloy 1 (Ag-80 at.% Al-10 at.% Au). Three regions with different contrasts can be observed in Figure 1a. The composition of the dark region is Ag-93.01 at.% Al-1.66 at.% Au, which is likely to be the (Al) phase [11]. The composition of the light region is Ag-69.60 at.% Al-27.05 at.% Au, which is likely to be the AuAl_2_ phase [11]. Han et al. also found a small shape with a light color with a phase composition atomic ratio Au to Al close to 1:2, which is determined as AuAl_2_ [16]. The composition of the gray region is Ag-39.57 at.% Al-2.92 at.% Au, which is likely to be the Ag_2_Al phase [10]. Figure 1b presents the XRD pattern of Alloy 1 and shows the diffraction peaks of the (Al), AuAl_2_, and Ag_2_Al phases [15]. This result is consistent with the SEM/EDS analysis. The EDS and XRD results indicate that the equilibrium phases of Alloy 1, aged at 450 °C, are in the triangle region composed of the (Al), AuAl_2_, and Ag_2_Al phases. Similar results were found for Alloys 2, 3, and 4, which show that the three-phase region is composed of the (Al), AuAl_2_, and Ag_2_Al phases. Figure 2a,b show a BEI micrograph and XRD pattern, respectively, for Alloy 6 (Ag-50 at.% Al-40 at.% Au) aged at 450 °C for 1440 h. Three phases were identified in this alloy, including Ag-62.34 at.% Al-35.18 at.% Au as the AuAl_2_ phase (dark region), Ag-44.90 at.% Al-52.77 at.% Au as the AuAl phase (bright region), and the Au_4_Al phase (gray region) composed of Ag-25.25 at.% Al-39.96 at.% Au [12]. The XRD result, as presented in Figure 2b, also confirmed the presence of the AuAl_2_, Au_4_Al, and AuAl phases in the ternary Alloy 6. Figure 3a,b show the BEI micrograph of the Ag-40 at.% Al-20 at.% Au alloy (Alloy 8) aged at 450 °C for 1440 h, respectively. Two phases were observed, including a dark region composed of Ag-23.11 at.% Al-16.55 at.% Au as the Au_4_Al phase and a bright region composed of Ag-64.72 at.% Al-24.59 at.% Au as the AuAl_2_ phase [9]. The XRD result, as presented in Figure 3b, also confirmed the presence of the Au_4_Al and AuAl_2_ phases in the ternary Alloy 8. Because the Ag-Au phase diagram is an isomorphous system, the considerable solubility of the Ag atoms can be incorporated into the Au sublattice in the Au_4_Al phase. This result was similar to the isothermal section of the Ag-Al-Au ternary system at 500 °C, as reported by Prince et al. [13]. These experiments aim to understand the interactions between these materials at elevated temperatures, which is relevant to electronic packaging applications. Similar phase composition results were found in Alloys 5 and 10 composed of the AuAl_2_ and Au_4_Al phases, as presented in Table 1.

**Figure 4:**
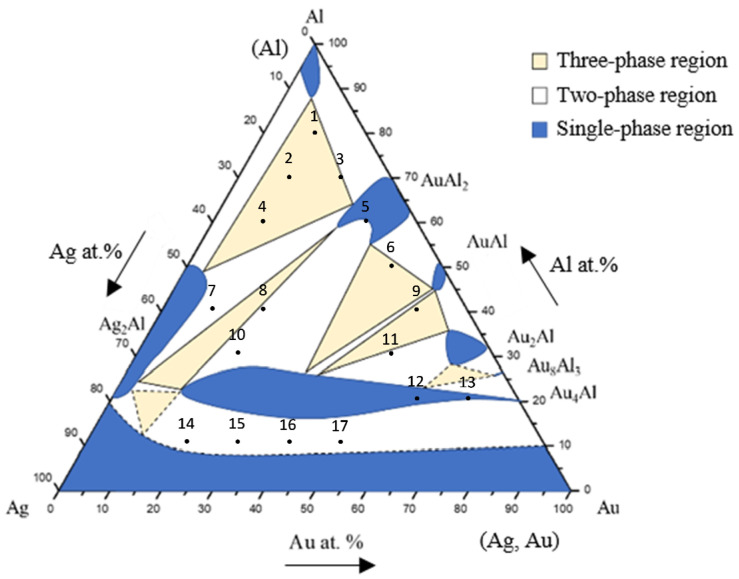
Isothermal section of the Ag-Al-Au ternary system at 450 °C. The solid lines depict the experimental results of the equilibrated phase, and the dashed lines depict the estimated results.

Based on the SEM-EDS experimental compositions summarized in Table 1 and the XRD results, the isothermal section of the Ag-Al-Au ternary system at 450 °C was established, as shown in Figure 4. The solid lines indicate the experimental results of the equilibrated phase, and the dashed lines indicate the estimated results phase based on the principle of phase equilibria and experimental data. The isothermal section of the Ag-Al-Au ternary system at 450 °C was composed of seven single-phase regions, thirteen two-phase regions, and six three-phase regions without any ternary compounds found based on the experimental results. The experimental results also show that the Au_4_Al phase had considerable solubility of the Ag atoms replacing the Au sublattices. The reason for this might be that Ag and Au can easily form a continuous solid solution. It seems that the Ag and Au atoms can easily substitute each other. The forming of a substitutional (continuous) solid solution in the entire alloy composition is a result of this effect.

### 3.2. Interfacial Reaction in the Au-25Ag/Al Reaction Couple at 450 °C

**Figure 5:**
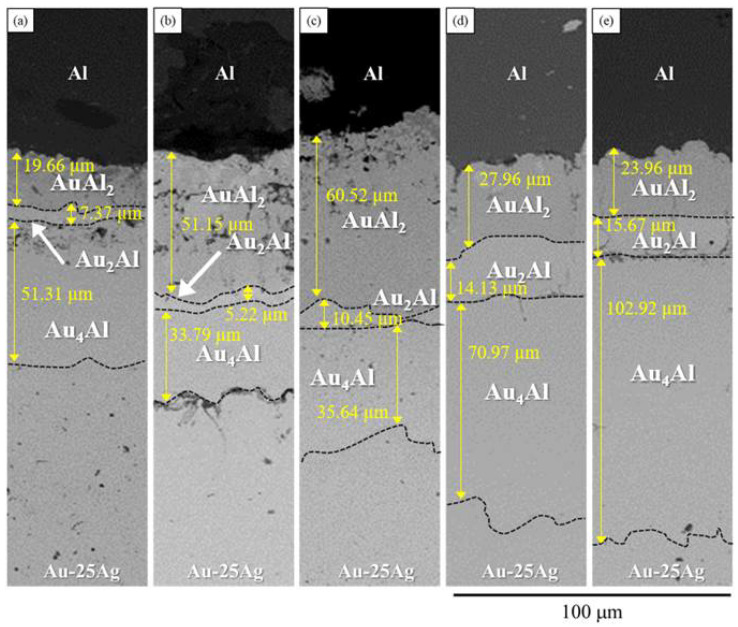
BEI micrograph of the Au-25Ag/Al reaction couples reacted at 450 °C for (**a**) 240; (**b**) 480; (**c**) 600; (**d**) 1000; (**e**) 1500 h.

With the progress of the thermal aging process, the atoms at the metallic interface interdiffused and reached the local thermodynamic equilibrium at the interface. The formation of intermetallic phases at the interface between two materials, such as Au-Ag/Al, is governed by various factors, including the local thermodynamic conditions, the composition of the materials, and the kinetics of the interdiffusion processes. Therefore, the IMC formed at the interface. Figure 5a shows the BEI micrograph of the Au-25Ag/Al reaction couple reacted at 450 °C for 240 h. Three-layer structures were observed at the interface. Using the EDS composition analysis, the compositions from the upper Al layer to the lower Au-25Ag alloy were Al-32.8 at.% Au, Ag-33.7 at.% Al-66.1 at.% Au, and Ag-23.5 at.% Al-45.2 at.% Au. According to the Ag-Al [11] and Al-Au [12] binary phase diagrams and the isothermal section, as established in Section 3.1, these layers, from the Al layer to the Au-25Ag alloy, were likely to be the AuAl_2_, Au_2_Al, and the Au_4_Al phases [12]. When the reaction time increased to 480 h, as shown in Figure 5b, the layered IMCs from the top to bottom consisted of these three Au-Al binary phases of the AuAl_2_, Au_2_Al, and Au_4_Al phases. A similar result was found in the Ag-Au-Pd/Al system reported by Guo et al. [17]. Although the thickness of each IMC, as shown in Figure 5a,b, increased significantly, no new IMC formed at the interface. As the reaction time progresses, the composition and thickness of these layers change, with the layered structure still present, but with increased thickness. The layers are still consistent with the AuAl_2_, Au_2_Al, and Au_4_Al phases. When the reaction time further increased to 600 h, 1000 h, and 1500 h (Figure 5c–e), the IMC formed at the interface consisted of the three binary Au-Al phases of the AuAl_2_, Au_2_Al, and Au_4_Al phases. However, the thickness of the first layer of the AuAl_2_ phase decreased and the thickness of the Au_2_Al and Au_4_Al phases increased when the reaction time was longer than 600 h and increased with the prolonged reaction time. Based on the measurement, the total IMC thickness increased from approximately 78 to 142 μm. The composition analysis suggests that the first layer next to the Al substrate is the AuAl_2_ phase. Therefore, in this case, the first phase to form at the interface is the AuAl_2_ phase.

**Figure 6:**
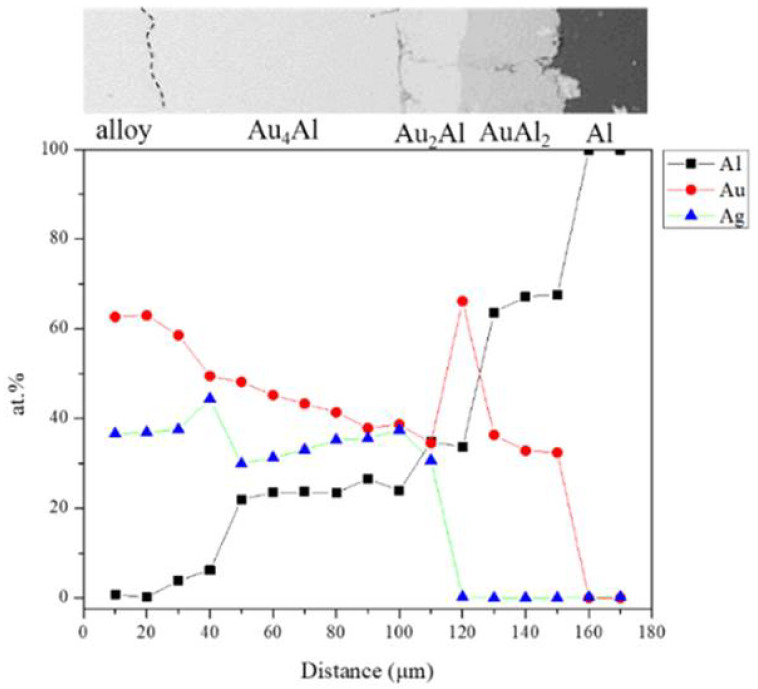
EPMA line scanning compositional profile of the Au-25Ag/Al reaction couples reacted at 450 °C for 600 h.

**Figure 7:**
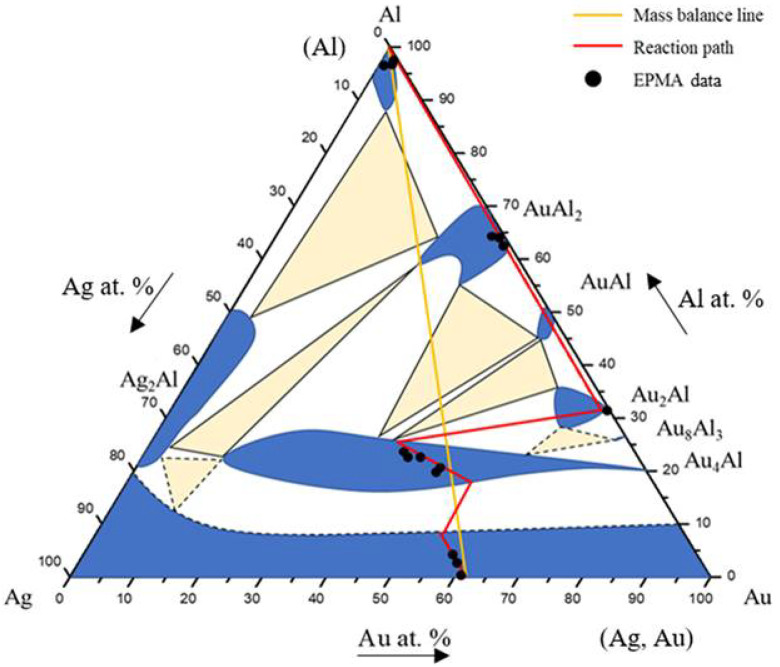
Reaction path of the Au-25Ag/Al reaction couples determined using EPMA line scanning superimposed with the experimental isothermal section at 450 °C.

Figure 6 shows the EPMA line scanning compositional profile of the Au-25Ag/Al (Au-37.8 at.% Ag) reaction couple reacted at 450 °C for 600 h. As shown in Figure 6, the AuAl_2_, Au_2_Al, and Au_4_Al phases can clearly be observed at the interfacial region. Figure 6 also reveals the Au_4_Al phase with a considerable Ag solubility in it. These EPMA quantitative analyses were also plotted on the 450 °C isothermal section of the Ag-Al-Au ternary phase diagram, as shown in Figure 7. The diffusion reaction path was Al/AuAl_2_/Au_2_Al/Au_4_Al/Au-25Ag alloy in the Au-25Ag/Al couples reacted at 450 °C.

### 3.3. Interfacial Reactions in the Au-50Ag/Al Reaction Couple

**Figure 8:**
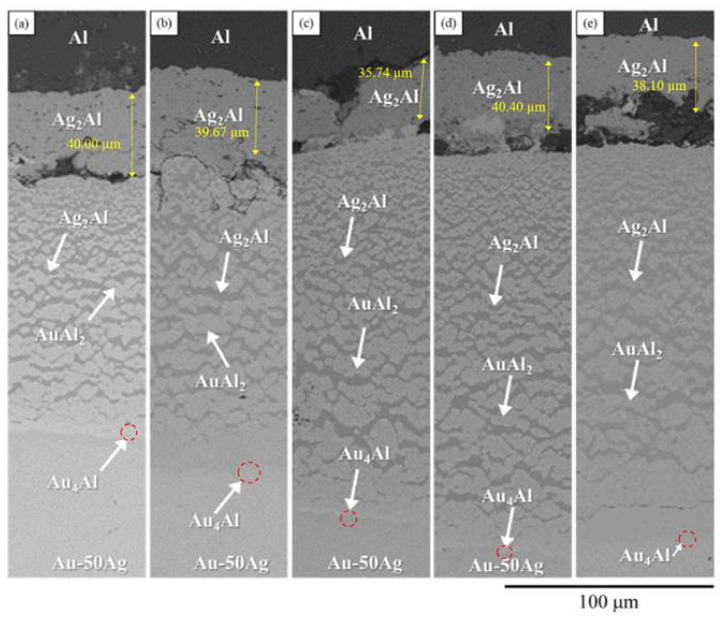
BEI micrograph of the Au-50Ag/Al reaction couples reacted at 450 °C for (**a**) 240; (**b**) 480; (**c**) 600; (**d**) 1000; (**e**) 1500 h.

A BEI micrograph of the Au-50Ag/Al reaction couple reacted at 450 °C for 240 h is shown in Figure 8a. We found that the multiple-layered structures formed from the upper Al layer to the lower Au-Ag alloy layer consisted of a layered-type Ag_2_Al phase [10] composed of Ag-41.4 at.% Al in the first layer of a mixture composed of the Ag_2_Al and AuAl_2_ (Ag-66.2 at.%Al-32.5 at.% Au) phase [8]. The continuous last layer had a unique composition of Ag-18.9 at.%Al-25.8 at.%, where Au is likely to be the Au_4_Al phase [11] with the considerable solubility of the Ag atoms. When the reaction time was increased from 480 to 1500 h (Figure 8b–e), the IMCs from the top to the bottom consisted of the layered Ag_2_Al phase, the mixture of the Ag_2_Al and the AuAl_2_ phases, and the layered Au_4_Al phase. These results were similar to those reported by Cho et al. [1]. The Ag_2_Al layer near the Al layer did not show significant changes with an increase in the reaction time. We found that its thickness was approximately 40 μm for all of the conditions. The thickness of the mixture composed of the Ag_2_Al and AuAl_2_ phases increased from 113 to 176 μm. Meanwhile, the amount of total IMCs increased with the increase in the reaction time. 

**Figure 9:**
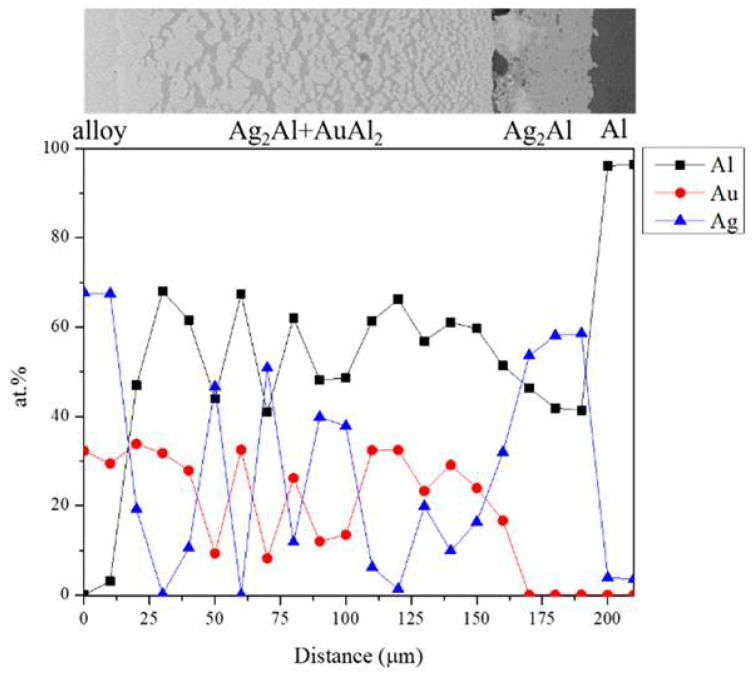
EPMA line scanning compositional profile of the Au-50Ag/Al reaction couples reacted at 450 °C for 600 h.

**Figure 10:**
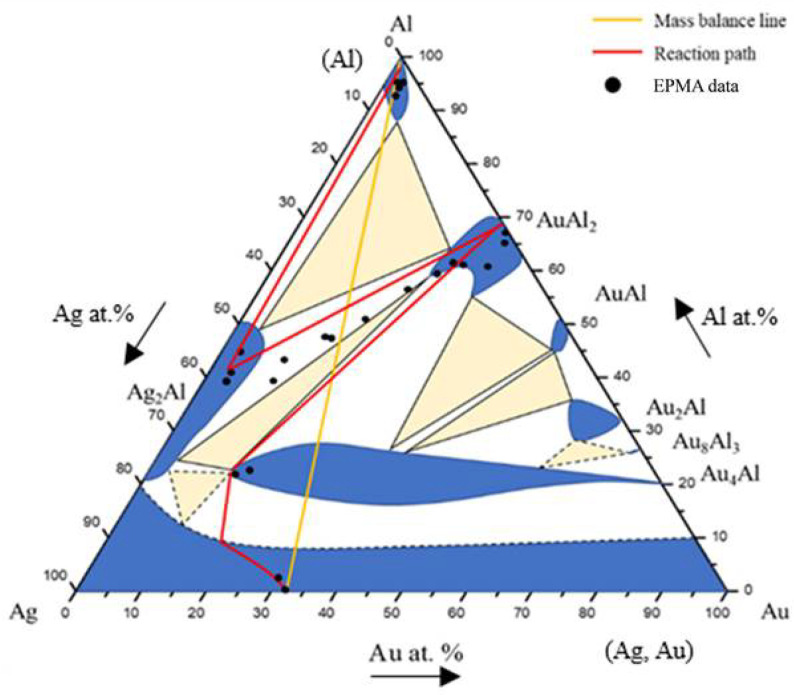
Reaction path of the Au-50Ag/Al reaction couples determined using EPMA line scanning superimposed with the experimental isothermal section at 450 °C.

Figure 9 shows the EPMA line-scan compositional profile of the Au-50Ag/Al (Au-64.6 at.% Ag) reaction couple reacted at 450 °C for 600 h. These EPMA quantitative analyses were also plotted on the isothermal section of the Ag-Al-Au ternary system at 450 °C, as shown in Figure 10. The wave-shaped structure at the Au_4_Al/Al interface can be observed in Figure 8c. The reaction path is a cross tie-line between the Au_4_Al and Al phases [18]. The reaction path in the Au-50Ag/Al couples isothermally annealed at 450°C was the Al/Ag_2_Al/Ag_2_Al+AuAl_2_/Au_4_Al/Au-50Ag alloy.

**Figure 11:**
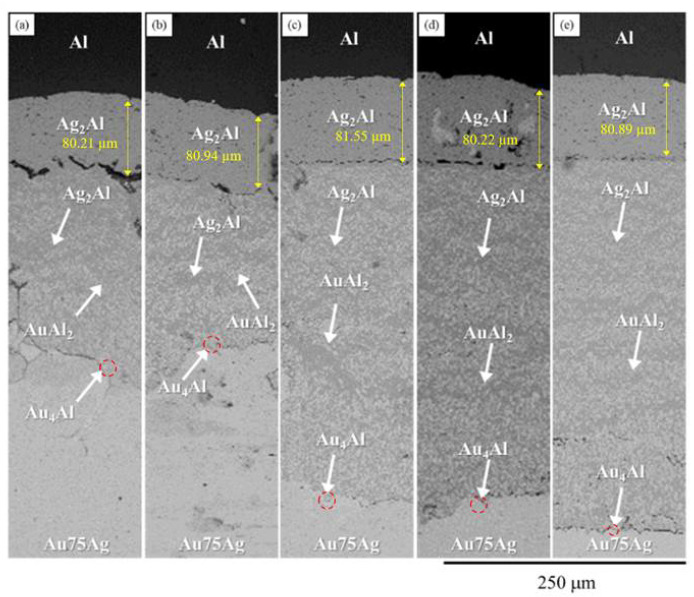
BEI micrograph of the Au-75Ag/Al reaction couples reacted at 450 °C for (**a**) 240, (**b**) 480, (**c**) 600, (**d**) 1000, and (**e**) 1500 h.

**Figure 12:**
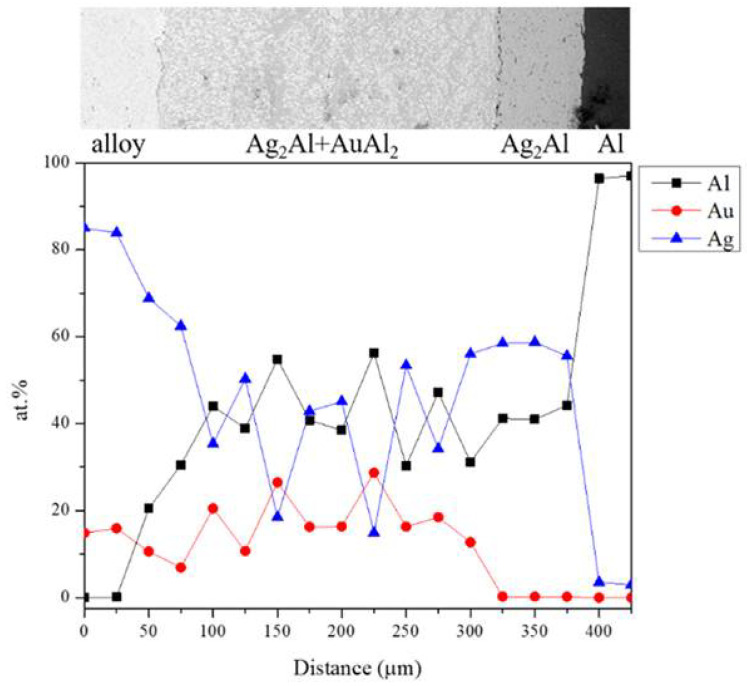
EPMA line scanning compositional profile of the Au-75Ag/Al reaction couples reacted at 450 °C for 600 h.

**Figure 13:**
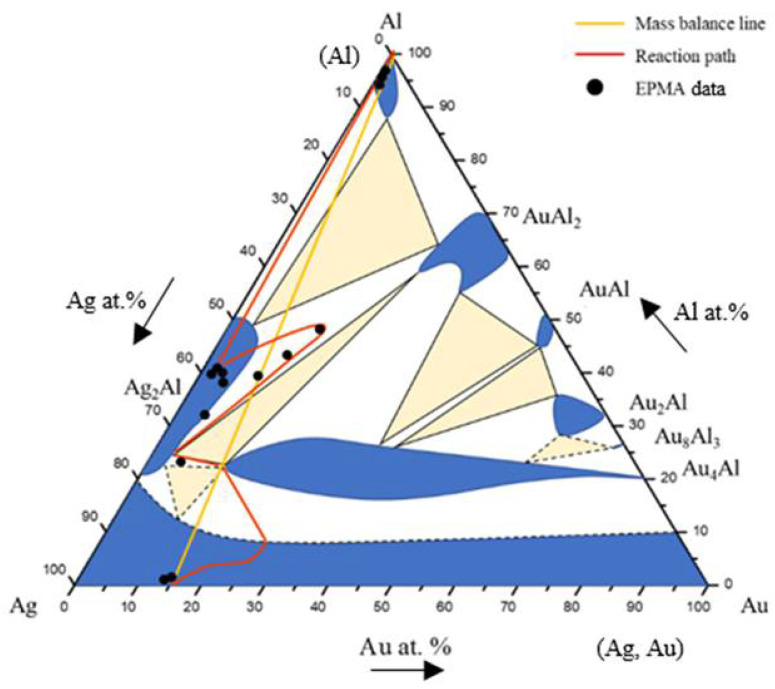
Reaction path of the Au-75Ag/Al reaction couples determined using EPMA line scanning superimposed with the experimental isothermal section at 450 °C.

A BEI micrograph of the Au-75Ag/Al reaction couple reacted at 450 °C for 240 h is shown in Figure 11a. Three-layer structures were observed at the interface which are Ag_2_Al, AuAl_2_, and the Au_4_Al phases. When the reaction time increased from 480 h to 1500 h (Figure 11b–e), no new IMC formed at the interface, but the IMC thickness increased. Figure 12 shows the EPMA line-scan compositional profile of the Au-75Ag/Al (Au-84.6 at.% Ag) reaction couple reacted at 450 °C for 600 h. These EPMA quantitative analyses were also plotted on the isothermal section of the Ag-Al-Au ternary system at 450 °C, as shown in Figure 13. Similar to that in the Au-50Ag/Al reaction couple, the reaction path in the Au-75Ag/Al couples reacted at 450 °C was the Al/Ag_2_Al/Ag_2_Al+AuAl_2_/Au_4_Al/Au-75Ag alloy. In this research, across Au-25Ag/Al, Au-50Ag/Al, and Au-75Ag/Al reaction couples, the predominant phase at the bonding interface is Au_4_Al, a consistent finding frequently observed in gold wire bonding originating from the Au/Al interface [19].

### 3.4. Ag Content Effect and Reaction Kinetics in the Au-xAg/Al Reaction Couples

The Ag content effect and the IMC growth kinetics in the Au-*x*Ag/Al reaction couples are discussed in this section. We found that the growth kinetics of the IMCs formed at the interfaces were altered by changing the Ag content in the Au-*x*Ag alloys. When the Ag content (*x*) was greater than 50 wt.%, no Au_2_Al phase was formed at the interface, as shown in Figure 8 and Figure 11. Based on the research reported by Yang et al. [20], we found the following results after comparing the mechanical properties between the AuAl_2_ and Au_2_Al phases. The Au-rich Au-Al IMC was softer and more ductile than the Al-rich Au-Al IMC to resist the propagation of cracks. In contrast, the Al-rich Au-Al IMC was more brittle, harder, and less ductile. Therefore, to improve the mechanical properties and the crack-propagation resistance, determining the optimum Ag content in the Au-*x*Ag alloy for the formation of the Au-rich Au_2_Al phase is necessary. The results presented in this study showed that the Au_2_Al phase was formed in the Au-25Ag/Al reaction couple. Therefore, the Au-*x*Ag alloys with *x* less than 50 wt.% is recommended based on both the experimental and phase diagram investigations. 

The Ag_2_Al phase serves as a barrier layer to inhibit the diffusion of Au atoms toward the Al layer to form the Au-Al IMCs [18]. In addition, the results of a study by Fu et al. [21] showed that the Young’s modulus of the Ag_2_Al phase was very close to that of the Au_2_Al phase. When the Ag_2_Al phase becomes the dominant IMC in the Ag-*x*Au/Al interface, it can improve the mechanical properties of the interface against crack propagation. However, when the Ag concentration was higher than 50 wt.%, the excess Ag_2_Al phase was formed at the interface, and it would degrade the mechanical performance of the interface [22,23].

**Figure 14:**
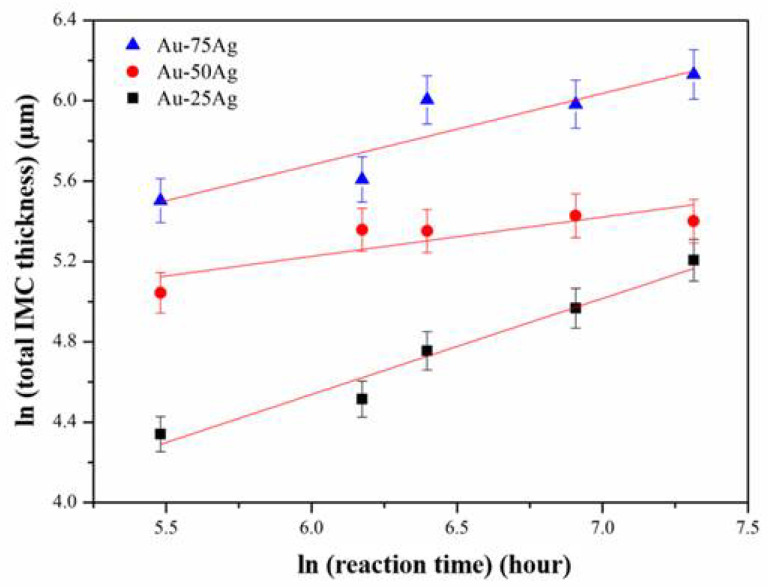
The natural logarithm plot of the total IMC thickness versus reaction time in the Au-*x*Ag/Al reaction couples.

The total thickness of the IMC formed at the interface can be represented by the following empirical power law: *x* = *k × t*^n^, where *x* is the total thickness of the IMC layer formed at the interface, *k* is the growth rate constant, n is the time exponent, and *t* is the reaction time. To evaluate the time exponent for the interfacial reaction in the Au-*x*Ag/Al reaction couples to obtain the IMC growth mechanism, the above equation was further converted into a logarithmic expression: *ln*(*x*) = n *× ln*(*t*) + *lnk*. As shown in Figure 14, the natural logarithm of the total IMC thickness showed a linear relationship with the natural logarithm of the reaction time in the Au-25Ag/Al, Au-50Ag/Al, and Au-75Ag/Al reaction couples. The total calculation of IMC thickness is derived from averaging measurements taken from more than five areas and subsequently determining the error. The slopes in Figure 14 are the n values in the empirical power law equation and are listed in Table 2. The n values of the Au-25Ag/Al, Au-50Ag/Al, and Au-75Ag/Al reaction couples were 0.9, 0.8, and 0.87, respectively. When the n value is equal to one, the IMC growth mechanism is controlled by the interfacial reaction [24,25]. Based on the results in Table 2, the n values for these three reaction couples were very close to one. This result reveals that the IMC growth mechanism in all reaction couples was controlled by the interfacial reaction.

## 4. Conclusions

The phase equilibria of the Ag-Al-Au ternary system at 450 °C were experimentally established. There were seven single-phase regions, thirteen two-phase regions, and six three-phase regions, with no ternary IMC formed in the isothermal section of the Ag-Al-Au ternary. Three IMCs, including the AuAl_2_, Au_2_Al, and Au_4_Al phases, formed in the Au-25Ag/Al reaction couple. When the Ag contents were increased to 50 and 75 wt.%, the Ag_2_Al, AuAl_2_, and Au_4_Al phases formed at the interface. The Au_4_Al phase showed a considerable solubility of the Ag element. These results revealed that the increase in the Ag content in the Au-*x*Ag alloy would make the AuAl_2_ phase convert to the Ag_2_Al phase. The total IMC thickness increased with the increase in the reaction time. When the Ag contents in the Au-*x*Ag alloy increased, the IMC layer composed of the Ag_2_Al and AuAl_2_ phases gradually transformed into the Ag_2_Al phase, and the Ag_2_Al phase became the major phase. The reaction paths for the Au-25Ag/Al, Au-50Ag/Al, and Au-75Ag/Al reaction couples were determined to be Al/AuAl_2_/Au_2_Al/Au_4_Al/Au-25Ag, Al/Ag_2_Al/Ag_2_Al+AuAl_2_/Au_4_Al/Au-50Ag alloy, and Al/Ag_2_Al/Ag_2_Al+AuAl_2_/Au_4_Al/Au-75Ag alloy, respectively. The IMC growth mechanism was the reaction controlled in these three reaction couples.

## Figures and Tables

**Table 1:** Phase equilibria of the Ag-Al-Au ternary systems with different compositions aged at 450 °C for 1440 h.

Alloy No.	Alloy Composition (at.%)	Phase in Equilibrium	Phase Composition (at.%)		
			Ag	Al	Au
1	Ag-80Al-10Au	AuAl_2_	3.35	69.60	27.05
		Ag_2_Al	57.51	39.57	2.98
		Al	5.26	93.08	1.66
2	Ag-70Al-10Au	AuAl_2_	9.84	66.76	23.44
		Ag_2_Al	52.22	44.95	2.83
		Al	6.67	90.40	2.93
3	Ag-70Al-20Au	AuAl_2_	2.01	67.11	30.88
		Ag_2_Al	47.73	46.95	5.32
		Al	3.64	93.21	3.15
4	Ag-60Al-10Au	AuAl_2_	13.17	56.81	30.02
		Ag_2_Al	53.15	43.26	3.59
		Al	6.5	90.83	2.67
5	Ag-60Al-30Au	Au_4_Al	51.71	27.12	21.17
		AuAl_2_	3.17	64.92	31.91
6	Ag-50Al-40Au	AuAl	2.33	44.9	52.77
		Au_4_Al	34.79	25.25	39.96
		AuAl_2_	2.48	62.34	35.18
7	Ag-40Al-10Au	AuAl_2_	17.32	59.39	23.29
		Ag_2_Al	65.23	30.37	4.4
8	Ag-40Al-20Au	Au_4_Al	60.34	23.11	16.55
		AuAl_2_	10.69	64.72	24.59
9	Ag-40Al-50Au	Au_2_Al	6.49	37.69	55.82
		Au_4_Al	34.79	25.25	39.96
		AuAl	5.91	43.11	50.98
10	Ag-30Al-20Au	AuAl_2_	11.37	60.24	28.39
		Au_4_Al	60.28	19.61	20.11
11	Ag-30Al-50Au	Au_2_Al	8.45	30.10	61.45
		Au_4_Al	33.16	24.80	42.04
12	Ag-20Al-60Au	Au_4_Al	20.01	20.3	59.69
13	Ag-20Al-70Au	(Ag, Au)	17.52	10.06	72.42
		Au_4_Al	19.00	19.23	61.77
14	Ag-10Al-20Au	(Ag, Au)	75.78	3.89	20.34
		Au_4_Al	51.73	19.31	28.97
15	Ag-10Al-30Au	(Ag, Au)	68.09	4.85	27.06
		Au_4_Al	47.58	15.88	36.54
16	Ag-10Al-40Au	(Ag, Au)	56.51	6.1	37.39
		Au_4_Al	33.16	20.71	46.13
17	Ag-10Al-50Au	(Ag, Au)	42.48	7.59	49.94
		Au_4_Al	30.3	18.56	51.14

**Table 2:** The n value of each Au-*x*Ag/Al reaction couple.

Reaction Couple	n Value
Au-25Ag	0.90
Au-50Ag	0.80
Au-75Ag	0.87

## Data Availability

All data generated or analyzed during this study are provided in this manuscript.
